# *N*-(4-Methyl­phenyl­sulfon­yl)-3-nitro­benzamide

**DOI:** 10.1107/S1600536814001317

**Published:** 2014-01-22

**Authors:** S. Sreenivasa, M. S. Nanjundaswamy, A. G. Sudha, K. J. Pampa, N. K. Lokanath, P. A. Suchetan

**Affiliations:** a Department of Studies and Research in Chemistry, Tumkur University, Tumkur, Karnataka 572 103, India; b Department of Chemistry, AVK College for Women, Davangere-2, India; c University College of Science, Tumkur University, Tumkur, India; d Department of Studies in Microbiology, University of Mysore, Manasagangotri, Mysore, India; e Department of Studies in Physics, University of Mysore, Manasagangotri, Mysore, India; f Department of Studies and Research in Chemistry, U.C.S., Tumkur University, Tumkur, Karnataka 572 103, India

## Abstract

In the title compound, C_14_H_12_N_2_O_5_S, the dihedral angle between the aromatic rings is 86.29 (1)° and the conformation between the C=O bond of the amide group and the *meta*-NO_2_ group is *syn*. The C—S—N—C torsion angle is −65.87 (19)° and the mol­ecule has an L-shaped conformation. In the crystal, the mol­ecules are connected into inversion dimers through pairs of N—H⋯O hydrogen bonds and C—H⋯O inter­actions forming *R*_2_^2^(8) and *R*_2_^2^(14) loops, respectively. The dimers are connected by further C—H⋯O inter­actions, thereby forming (100) sheets.

## Related literature   

For related structures see: Suchetan *et al.* (2010 ▶, 2011 ▶, 2012 ▶).
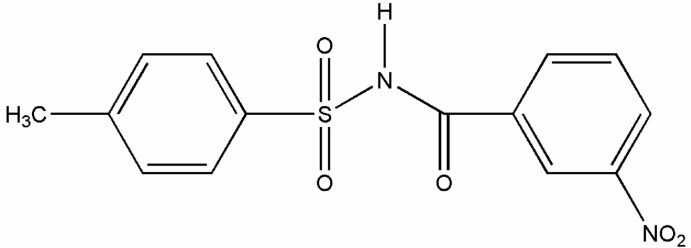


## Experimental   

### 

#### Crystal data  

C_14_H_12_N_2_O_5_S
*M* *_r_* = 320.32
Monoclinic, 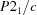

*a* = 4.9736 (5) Å
*b* = 23.245 (2) Å
*c* = 12.7197 (11) Å
β = 100.820 (4)°
*V* = 1444.4 (2) Å^3^
*Z* = 4
Cu *K*α radiation
μ = 2.24 mm^−1^
*T* = 293 K
0.39 × 0.29 × 0.20 mm

#### Data collection  

Bruker APEXII diffractometer
Absorption correction: multi-scan (*SADABS*; Bruker, 2009 ▶) *T*_min_ = 0.481, *T*_max_ = 0.638
12115 measured reflections
2378 independent reflections
2053 reflections with *I* > 2σ(*I*)
*R*_int_ = 0.035

#### Refinement  

*R*[*F* ^2^ > 2σ(*F* ^2^)] = 0.039
*wR*(*F* ^2^) = 0.114
*S* = 1.06
2378 reflections
204 parameters
H atoms treated by a mixture of independent and constrained refinement
Δρ_max_ = 0.20 e Å^−3^
Δρ_min_ = −0.23 e Å^−3^

### 

Data collection: *APEX2* (Bruker, 2009 ▶); cell refinement: *APEX2* and *SAINT-Plus* (Bruker, 2009 ▶); data reduction: *SAINT-Plus* and *XPREP* (Bruker, 2009 ▶); program(s) used to solve structure: *SHELXS97* (Sheldrick, 2008 ▶); program(s) used to refine structure: *SHELXL97* (Sheldrick, 2008 ▶); molecular graphics: *Mercury* (Macrae *et al.*, 2008 ▶); software used to prepare material for publication: *SHELXL97*.

## Supplementary Material

Crystal structure: contains datablock(s) I. DOI: 10.1107/S1600536814001317/hb7184sup1.cif

Structure factors: contains datablock(s) I. DOI: 10.1107/S1600536814001317/hb7184Isup2.hkl

Supporting information file. DOI: 10.1107/S1600536814001317/hb7184Isup3.cml

CCDC reference:

Additional supporting information:  crystallographic information; 3D view; checkCIF report

## Figures and Tables

**Table 1:** Hydrogen-bond geometry (Å, °)

*D*—H⋯*A*	*D*—H	H⋯*A*	*D*⋯*A*	*D*—H⋯*A*
N1—H*N*1⋯O2^i^	0.80 (3)	2.14 (3)	2.927 (3)	167
C13—H13⋯O2^i^	0.93	2.59	3.333 (3)	137
C3—H3⋯O4^ii^	0.93	2.58	3.459 (3)	155

Symmetry codes: (i) 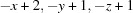; (ii) 
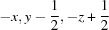
.

**Fig. 1.**
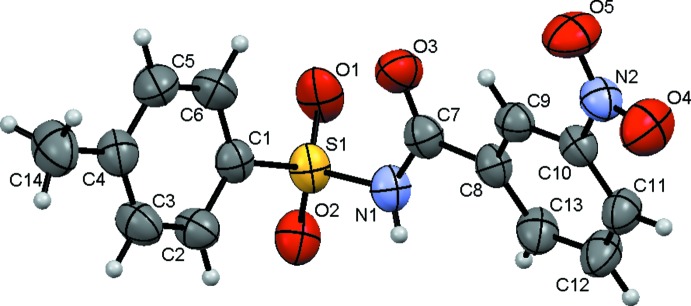
Molecular structure of the title compound, showing displacement ellipsoids at the 50% probability level.

**Fig. 2.**
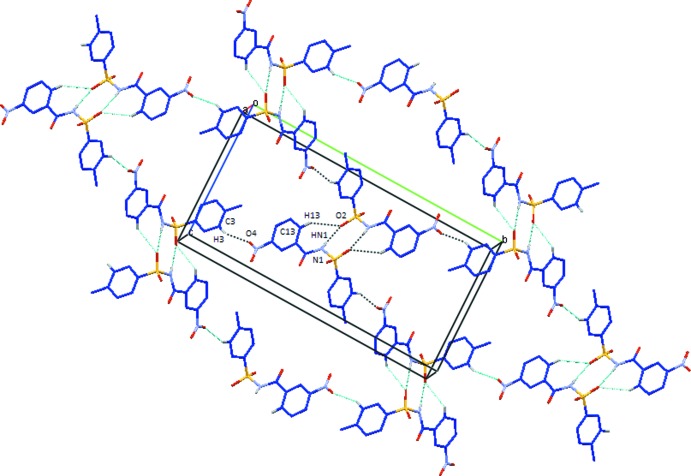
Formation of sheets in the crystal structure.

## supplementary crystallographic information

### 1. Introduction

As a part of our continued efforts to study the crystal structures of N-(aroyl)-aryl­sulfonamides (Suchetan *et al.*, 2010, 2011, 2012), we report here the crystal structure of the title compound (I) (Fig 1).

### 2.1. Synthesis and crystallization

The title compound (I) was prepared by refluxing a mixture of 3-nitro­benzoic acid, 4-methyl­benzene­sulfonamide and phospho­rous oxychloride (POCl_3_) for 2 h on a water bath. The resultant mixture was cooled and poured into ice cold water. The solid obtained was filtered and washed thoroughly with water and then dissolved in sodium bicarbonate solution. The compound was later reprecipitated by acidifying the filtered solution with dilute HCl. The compound obtained was filtered and later dried (Melting point: 459 K).

Colorless prisms of (I) were obtained from a slow evaporation of its aqueous methano­lic solution at room temperature.

### 2.2. Refinement

The H atom of the NH group was located in a difference map and later refined freely. The other H atoms were positioned with idealized geometry using a riding model with C—H = 0.93-0.96 Å. All H atoms were refined with isotropic displacement parameters (set to 1.2-1.5 times of the Ueq of the parent atom).

### 3. Results and discussion

In I, the dihedral angle between the two aromatic rings is 86.29 (1)°. Compared to this, the dihedral angle is 79.4 (1)° in N-(4-methyl­phenyl­sulfonyl)-benzamide (II) (Suchetan *et al.*, 2010), 89.8 (1)° in N-(4-methyl­phenyl­sulfonyl)-4-nitro­benzamide (III) (Suchetan *et al.*, 2011) and 86.9 (2)° in N-(phenyl­sulfonyl)-3-nitro­benzamide (IV) (Suchetan *et al.*, 2012). Thus, introducing a nitro group into the benzoyl ring results in an increase of the dihedral angle between the aromatic rings. The conformation between the N—H bond and the *meta*-NO_2_ group is *anti* in contrast to the *syn* conformation observed in IV (Suchetan *et al.*, 2012). The molecule is twisted at the S atom, the dihedral angle between the planes defined by the S—N—C=O segment in the central chain and the sulfonyl benzene rings being 79.16 (1)°.

In the crystal structure, the molecules are connected into inversion dimers through N1—HN1···O2 hydrogen bonds (Table 1, Figure 2) and C13—H13···O2 inter­actions forming R_2_^2^(8) and R_2_^2^(14) ring motifs respectively. These dimers are further connected into C(12) chains through C3—H3···O4 inter­actions forming sheets (Figure 2).

### Crystal data

**Table:**

C_14_H_12_N_2_O_5_S	Prism
*M**_r_* = 320.32	*D*_x_ = 1.473 Mg m^−^^3^
Monoclinic, *P*2_1_/*c*	Melting point: 459 K
Hall symbol: -P 2ybc	Cu *K*α radiation, λ = 1.54178 Å
*a* = 4.9736 (5) Å	Cell parameters from 1127 reflections
*b* = 23.245 (2) Å	θ = 3.8–64.5°
*c* = 12.7197 (11) Å	µ = 2.24 mm^−^^1^
β = 100.820 (4)°	*T* = 293 K
*V* = 1444.4 (2) Å^3^	Prism, colourless
*Z* = 4	0.39 × 0.29 × 0.20 mm
*F*(000) = 664	

### Data collection

**Table:**

Bruker APEXII diffractometer	2053 reflections with *I* > 2σ(*I*)
Radiation source: fine-focus sealed tube	*R*_int_ = 0.035
Graphite monochromator	θ_max_ = 64.5°, θ_min_ = 3.8°
phi and φ scans	*h* = −5→4
Absorption correction: multi-scan (*SADABS*; Bruker, 2009)	*k* = −26→26
*T*_min_ = 0.481, *T*_max_ = 0.638	*l* = −14→14
12115 measured reflections	2 standard reflections every 1 reflections
2378 independent reflections	intensity decay: 1%

### Refinement

**Table:**

Refinement on *F*^2^	Primary atom site location: structure-invariant direct methods
Least-squares matrix: full	Secondary atom site location: difference Fourier map
*R*[*F*^2^ > 2σ(*F*^2^)] = 0.039	Hydrogen site location: inferred from neighbouring sites
*wR*(*F*^2^) = 0.114	H atoms treated by a mixture of independent and constrained refinement
*S* = 1.06	*w* = 1/[σ^2^(*F*_o_^2^) + (0.0621*P*)^2^ + 0.3691*P*] where *P* = (*F*_o_^2^ + 2*F*_c_^2^)/3
2378 reflections	(Δ/σ)_max_ < 0.001
204 parameters	Δρ_max_ = 0.20 e Å^−^^3^
0 restraints	Δρ_min_ = −0.23 e Å^−^^3^

### Special details

**Table:**

Geometry. All s.u.'s (except the s.u. in the dihedral angle between two l.s. planes) are estimated using the full covariance matrix. The cell s.u.'s are taken into account individually in the estimation of s.u.'s in distances, angles and torsion angles; correlations between s.u.'s in cell parameters are only used when they are defined by crystal symmetry. An approximate (isotropic) treatment of cell s.u.'s is used for estimating s.u.'s involving l.s. planes.
Refinement. Refinement of *F*^2^ against ALL reflections. The weighted *R*-factor *wR* and goodness of fit *S* are based on *F*^2^, conventional *R*-factors *R* are based on *F*, with *F* set to zero for negative *F*^2^. The threshold expression of *F*^2^ > 2σ(*F*^2^) is used only for calculating *R*-factors(gt) *etc*. and is not relevant to the choice of reflections for refinement. *R*-factors based on *F*^2^ are statistically about twice as large as those based on *F*, and *R*- factors based on ALL data will be even larger.

### Fractional atomic coordinates and isotropic or equivalent isotropic displacement parameters (Å^2^)

**Table:**

	*x*	*y*	*z*	*U*_iso_*/*U*_eq_	
S1	1.10155 (10)	0.50190 (2)	0.32699 (4)	0.0546 (2)	
C8	0.6073 (4)	0.63811 (8)	0.33442 (15)	0.0481 (5)	
C9	0.4226 (4)	0.66836 (8)	0.25927 (16)	0.0481 (5)	
H9	0.4108	0.6615	0.1865	0.058*	
O2	1.1994 (3)	0.47252 (7)	0.42557 (13)	0.0667 (4)	
N2	0.0629 (4)	0.74082 (8)	0.21424 (15)	0.0570 (4)	
O1	1.2908 (3)	0.52526 (7)	0.26821 (15)	0.0720 (5)	
N1	0.9164 (4)	0.55519 (7)	0.36288 (16)	0.0534 (4)	
C10	0.2572 (4)	0.70859 (8)	0.29425 (15)	0.0471 (5)	
O3	0.8066 (3)	0.59550 (7)	0.19878 (13)	0.0695 (5)	
O4	−0.1159 (4)	0.76822 (9)	0.24399 (15)	0.0891 (6)	
O5	0.0900 (4)	0.73809 (9)	0.12131 (13)	0.0853 (6)	
C4	0.4935 (5)	0.38740 (9)	0.11605 (18)	0.0593 (5)	
C3	0.5450 (5)	0.38158 (10)	0.22531 (18)	0.0646 (6)	
H3	0.4508	0.3537	0.2563	0.077*	
C7	0.7819 (4)	0.59512 (9)	0.29113 (17)	0.0518 (5)	
C5	0.6344 (5)	0.42936 (10)	0.07092 (18)	0.0680 (6)	
H5	0.6014	0.4341	−0.0030	0.082*	
C11	0.2669 (5)	0.71991 (9)	0.40117 (17)	0.0583 (5)	
H11	0.1494	0.7467	0.4229	0.070*	
C1	0.8710 (4)	0.45716 (8)	0.24416 (16)	0.0490 (5)	
C6	0.8239 (5)	0.46431 (10)	0.13467 (19)	0.0630 (6)	
H6	0.9182	0.4923	0.1039	0.076*	
C13	0.6243 (5)	0.64965 (9)	0.44227 (17)	0.0602 (6)	
H13	0.7500	0.6298	0.4928	0.072*	
C2	0.7316 (5)	0.41580 (10)	0.29029 (17)	0.0588 (5)	
H2	0.7635	0.4111	0.3642	0.071*	
C12	0.4558 (5)	0.69042 (10)	0.47530 (18)	0.0669 (6)	
H12	0.4696	0.6981	0.5479	0.080*	
C14	0.2854 (6)	0.34997 (12)	0.0463 (2)	0.0845 (8)	
H14A	0.1814	0.3292	0.0903	0.127*	
H14B	0.3776	0.3232	0.0078	0.127*	
H14C	0.1647	0.3736	−0.0035	0.127*	
HN1	0.860 (5)	0.5495 (10)	0.417 (2)	0.062 (8)*	

### Atomic displacement parameters (Å^2^)

**Table:**

	*U*^11^	*U*^22^	*U*^33^	*U*^12^	*U*^13^	*U*^23^
S1	0.0368 (3)	0.0518 (3)	0.0732 (4)	0.0021 (2)	0.0053 (2)	−0.0047 (2)
C8	0.0445 (11)	0.0416 (10)	0.0566 (11)	−0.0023 (8)	0.0052 (9)	−0.0015 (8)
C9	0.0469 (11)	0.0478 (11)	0.0488 (10)	−0.0051 (9)	0.0071 (8)	−0.0001 (8)
O2	0.0538 (9)	0.0660 (10)	0.0726 (10)	0.0159 (7)	−0.0079 (7)	−0.0034 (8)
N2	0.0582 (11)	0.0534 (10)	0.0563 (11)	0.0032 (9)	0.0024 (8)	0.0041 (8)
O1	0.0428 (8)	0.0662 (10)	0.1106 (13)	−0.0065 (7)	0.0238 (8)	−0.0066 (9)
N1	0.0444 (10)	0.0513 (10)	0.0627 (11)	0.0046 (8)	0.0053 (8)	−0.0017 (8)
C10	0.0458 (11)	0.0423 (10)	0.0508 (11)	−0.0017 (8)	0.0027 (8)	0.0028 (8)
O3	0.0759 (11)	0.0679 (10)	0.0705 (11)	0.0121 (8)	0.0292 (8)	0.0079 (8)
O4	0.0871 (13)	0.0949 (14)	0.0821 (12)	0.0449 (11)	0.0077 (10)	0.0063 (10)
O5	0.0957 (14)	0.1043 (14)	0.0529 (10)	0.0235 (11)	0.0063 (9)	0.0148 (9)
C4	0.0611 (13)	0.0514 (12)	0.0619 (13)	0.0011 (10)	0.0025 (10)	−0.0020 (10)
C3	0.0647 (14)	0.0599 (13)	0.0669 (14)	−0.0144 (11)	0.0066 (11)	0.0075 (11)
C7	0.0448 (11)	0.0488 (11)	0.0612 (13)	−0.0026 (9)	0.0084 (9)	−0.0004 (9)
C5	0.0866 (17)	0.0651 (14)	0.0516 (12)	−0.0038 (12)	0.0111 (11)	−0.0008 (10)
C11	0.0665 (14)	0.0519 (11)	0.0551 (12)	0.0116 (10)	0.0082 (10)	−0.0036 (9)
C1	0.0414 (11)	0.0471 (10)	0.0587 (12)	0.0034 (8)	0.0100 (8)	−0.0014 (9)
C6	0.0686 (15)	0.0589 (13)	0.0647 (13)	−0.0080 (11)	0.0207 (11)	0.0028 (10)
C13	0.0670 (14)	0.0536 (12)	0.0540 (12)	0.0093 (10)	−0.0041 (10)	0.0001 (9)
C2	0.0591 (13)	0.0605 (13)	0.0546 (12)	−0.0081 (10)	0.0054 (10)	0.0051 (10)
C12	0.0871 (17)	0.0623 (14)	0.0471 (11)	0.0166 (12)	0.0017 (11)	−0.0057 (10)
C14	0.096 (2)	0.0701 (16)	0.0780 (17)	−0.0173 (14)	−0.0082 (14)	−0.0066 (13)

### Geometric parameters (Å, º)

**Table:**

S1—O1	1.4156 (17)	C4—C14	1.507 (3)
S1—O2	1.4305 (16)	C3—C2	1.375 (3)
S1—N1	1.6580 (18)	C3—H3	0.9300
S1—C1	1.747 (2)	C5—C6	1.385 (3)
C8—C13	1.385 (3)	C5—H5	0.9300
C8—C9	1.386 (3)	C11—C12	1.382 (3)
C8—C7	1.495 (3)	C11—H11	0.9300
C9—C10	1.373 (3)	C1—C6	1.378 (3)
C9—H9	0.9300	C1—C2	1.379 (3)
N2—O4	1.211 (2)	C6—H6	0.9300
N2—O5	1.216 (2)	C13—C12	1.382 (3)
N2—C10	1.470 (3)	C13—H13	0.9300
N1—C7	1.383 (3)	C2—H2	0.9300
N1—HN1	0.80 (2)	C12—H12	0.9300
C10—C11	1.377 (3)	C14—H14A	0.9600
O3—C7	1.204 (3)	C14—H14B	0.9600
C4—C3	1.372 (3)	C14—H14C	0.9600
C4—C5	1.387 (3)		
			
O1—S1—O2	119.71 (11)	N1—C7—C8	116.62 (19)
O1—S1—N1	108.51 (10)	C6—C5—C4	120.8 (2)
O2—S1—N1	103.33 (10)	C6—C5—H5	119.6
O1—S1—C1	109.63 (10)	C4—C5—H5	119.6
O2—S1—C1	108.74 (10)	C10—C11—C12	118.2 (2)
N1—S1—C1	105.96 (9)	C10—C11—H11	120.9
C13—C8—C9	119.62 (18)	C12—C11—H11	120.9
C13—C8—C7	124.23 (18)	C6—C1—C2	120.7 (2)
C9—C8—C7	116.14 (18)	C6—C1—S1	120.36 (16)
C10—C9—C8	118.78 (18)	C2—C1—S1	118.94 (16)
C10—C9—H9	120.6	C1—C6—C5	119.3 (2)
C8—C9—H9	120.6	C1—C6—H6	120.4
O4—N2—O5	123.57 (19)	C5—C6—H6	120.4
O4—N2—C10	118.53 (19)	C12—C13—C8	120.42 (19)
O5—N2—C10	117.89 (18)	C12—C13—H13	119.8
C7—N1—S1	123.00 (17)	C8—C13—H13	119.8
C7—N1—HN1	118.4 (18)	C3—C2—C1	119.0 (2)
S1—N1—HN1	114.5 (17)	C3—C2—H2	120.5
C9—C10—C11	122.56 (18)	C1—C2—H2	120.5
C9—C10—N2	118.58 (17)	C11—C12—C13	120.4 (2)
C11—C10—N2	118.86 (18)	C11—C12—H12	119.8
C3—C4—C5	118.4 (2)	C13—C12—H12	119.8
C3—C4—C14	121.1 (2)	C4—C14—H14A	109.5
C5—C4—C14	120.5 (2)	C4—C14—H14B	109.5
C4—C3—C2	121.9 (2)	H14A—C14—H14B	109.5
C4—C3—H3	119.0	C4—C14—H14C	109.5
C2—C3—H3	119.0	H14A—C14—H14C	109.5
O3—C7—N1	121.6 (2)	H14B—C14—H14C	109.5
O3—C7—C8	121.82 (19)		
			
C13—C8—C9—C10	−1.0 (3)	C14—C4—C5—C6	179.3 (2)
C7—C8—C9—C10	−179.78 (17)	C9—C10—C11—C12	1.7 (3)
O1—S1—N1—C7	51.79 (19)	N2—C10—C11—C12	−178.2 (2)
O2—S1—N1—C7	179.85 (17)	O1—S1—C1—C6	−23.4 (2)
C1—S1—N1—C7	−65.87 (19)	O2—S1—C1—C6	−155.95 (17)
C8—C9—C10—C11	−0.3 (3)	N1—S1—C1—C6	93.55 (19)
C8—C9—C10—N2	179.61 (17)	O1—S1—C1—C2	158.45 (17)
O4—N2—C10—C9	166.6 (2)	O2—S1—C1—C2	25.9 (2)
O5—N2—C10—C9	−12.9 (3)	N1—S1—C1—C2	−84.63 (19)
O4—N2—C10—C11	−13.5 (3)	C2—C1—C6—C5	−0.1 (3)
O5—N2—C10—C11	167.0 (2)	S1—C1—C6—C5	−178.20 (18)
C5—C4—C3—C2	−0.3 (4)	C4—C5—C6—C1	−0.2 (4)
C14—C4—C3—C2	−179.2 (2)	C9—C8—C13—C12	1.0 (3)
S1—N1—C7—O3	−4.9 (3)	C7—C8—C13—C12	179.6 (2)
S1—N1—C7—C8	175.83 (14)	C4—C3—C2—C1	0.0 (4)
C13—C8—C7—O3	−162.5 (2)	C6—C1—C2—C3	0.2 (3)
C9—C8—C7—O3	16.1 (3)	S1—C1—C2—C3	178.32 (18)
C13—C8—C7—N1	16.8 (3)	C10—C11—C12—C13	−1.7 (4)
C9—C8—C7—N1	−164.58 (18)	C8—C13—C12—C11	0.4 (4)
C3—C4—C5—C6	0.4 (4)		

### Hydrogen-bond geometry (Å, º)

**Table:**

*D*—H···*A*	*D*—H	H···*A*	*D*···*A*	*D*—H···*A*
N1—H*N*1···O2^i^	0.80 (3)	2.14 (3)	2.927 (3)	167
C13—H13···O2^i^	0.93	2.59	3.333 (3)	137
C3—H3···O4^ii^	0.93	2.58	3.459 (3)	155

Symmetry codes: (i) −*x*+2, −*y*+1, −*z*+1; (ii) −*x*, *y*−1/2, −*z*+1/2.
